# Genetic analysis reveals the genetic diversity and zoonotic potential of *Streptococcus dysgalactiae* isolates from sheep

**DOI:** 10.1038/s41598-025-87781-3

**Published:** 2025-01-25

**Authors:** Ilona Stefańska, Ewelina Kwiecień, Anna Didkowska, Magdalena Kizerwetter-Świda, Dorota Chrobak-Chmiel, Agnieszka Sałamaszyńska-Guz, Piotr Żmuda, Krzysztof Anusz, Magdalena Rzewuska

**Affiliations:** 1 Department of Preclinical Sciences, Institute of Veterinary Medicine, Warsaw University of Life Sciences, Ciszewskiego 8 St, 02-786 Warsaw, Poland; 2 Department of Food Hygiene and Public Health Protection, Institute of Veterinary Medicine, Warsaw University of Life Sciences, Nowoursynowska 159 St, 02-776 Warsaw, Poland; 3 University Centre of Veterinary Medicine, University of Agriculture in Krakow, Al. Mickiewicza 24/28, 30-059 Krakow, Poland

**Keywords:** Beta-hemolytic streptococci, Multilocus sequence typing, M protein gene (*emm*) typing, Sheep, *Streptococcus dysgalactiae*, Zoonotic pathogen, Microbiology, Bacteriology, Pathogens

## Abstract

*Streptococcus dysgalactiae* (*S. dysgalactiae* ) is a common pathogen of humans and various animals. However, the phylogenetic position of animal *S. dysgalactiae* isolates and their zoonotic potential remain unclear. Most molecular epidemiological studies explicate beta-hemolytic streptococci according to their MLST and M protein gene (*emm*) types. Although human *S. dysgalactiae* isolates are relatively well characterized, the data concerning animal isolates are scarce. Here, we report the molecular characteristics and antimicrobial resistance of *S. dysgalactiae* strains recovered from sheep and their genetic relationship with isolates from other animal hosts and humans. Overall, 11 PFGE pulsotypes, five MLST sequence types (STs), and two *emm* types were distinguished, with ST248 and *stL1376* being the most prevalent, indicating genetic diversity among tested 17 ovine isolates. Some isolates exhibited resistance to doxycycline (59%), erythromycin (6%), ciprofloxacin (6%), and trimethoprim/sulfamethoxazole (6%), harboring various resistance determinants. Phylogenetic analysis showed that studied ovine isolates grouped together with human *S. dysgalactiae* isolates from the cases of zoonotic infections. Moreover, some ovine isolates shared identical STs and *emm* gene sequences with human non-invasive and invasive *S. dysgalactiae* strains. These findings suggest a possible link between human and ovine isolates and indicate the zoonotic potential of this pathogen.

## Introduction

The species *Streptococcus dysgalactiae* (*S. dysgalactiae*) belongs to beta-hemolytic streptococci. Generally, these bacteria are considered opportunistic pathogens that can colonize various host species and cause various human and animal infections^1–3^. Molecular characterization of *S. dysgalactiae* strains showed that identical or closely related strains were isolated from humans and animals and supported the zoonotic transmission^4–6^. However, insufficient diagnostics of *S. dysgalactiae* infections, frequently without complete identification of beta-hemolytic streptococci to the species level, makes difficult the assessment of the pathogenicity as well as the zoonotic potential of these bacteria.

*S. dysgalactiae* consists of two subspecies, *S. dysgalactiae* subsp*. dysgalactiae* (SDSD) and *S. dysgalactiae* subsp*. equisimilis* (SDSE), but confusion about the characteristics of strains in each subspecies is common in the literature^2,7,8^. In 1996, Vandamme et al. defined subspecies and proposed the first classification^9^. According to this proposal, SDSE strains were associated with human diseases, while SDSD included strains of animal origin. Two years later, Vieira et al.^1^ restricted the SDSD subspecies only to bovine alpha- or gamma-hemolytic Lancefield group C strains, while the SDSE subspecies included all beta-hemolytic Lancefield group C, G, or L strains, isolated from both, humans or animals^1^. Although this classification has been widely used for years, many studies indicate that the proposed taxonomic division needs to be verified and more accurate^2,8,10,11^. The studies of the genetic relationship between *S. dysgalactiae* strains based on the multilocus sequence analysis (MLSA) showed that SDSE is beta-hemolytic and exclusively associated with humans, while SDSD strains demonstrating all types of hemolysis and are associated with various animal species^2,7^. Interestingly, the results of phylogenetic studies based on whole-genome sequencing of *S. dysgalactiae* strains from animal and human origins are inconsistent, and the subspecies classification of isolates from animals other than cattle is not established. Pinho et al.^7^ showed higher ANI (average nucleotide identity) values when comparing the genomes of equine isolates with human isolates than with the genome of the bovine strain SDSD ATCC® 27957^7^. Another ANI analysis using over 150 *S. dysgalactiae* isolates derived from human and various animal hosts indicated that bovine and ovine isolates formed one separate cluster and were classified as SDSD, while isolates from other animal hosts and humans formed a second cluster and should, therefore, be classified as SDSE^12^. In addition, the SDSE clade was divided into two subclusters, a human subcluster and a heterogeneous animal subcluster, which could be delineated in accordance with host species^12^. In contrast, the phylogenomic analysis of *S. dysgalactiae* strains conducted by Alves-Barroco et al.^8^ divided human and animal isolates into two clearly separated groups, corresponding to the subspecies SDSE and SDSD, respectively. Bovine SDSD isolates were grouped into a unique, distinct clade among animal cluster, while ovine strains were not analyzed^8^. All these observations show how crucial further research on the molecular characterization of strains of various origins is to clarify the classification of streptococci isolated from animals^2,7^. Given this taxonomic discordance, in order to avoid further confusion, in our paper we use the name *S. dysgalactiae* for strains isolated from animals other than cattle, without distinguishing between subspecies.

In sheep, *S. dysgalactiae* is one of the most important causative agents of outbreaks of infectious polyarthritis in lambs, which presents a high mortality rate and an important animal health issue in many countries. *S. dysgalactiae* infections in sheep can also be associated with clinical and subclinical mastitis, bacteriemia and meningoencephalitis^13–17^. These bacteria were among the two most common pathogens isolated from purulent or caseous lymphadenitis in sheep in Poland (34.7%, CI 95%: 21.7%-49.6%)^18^. *S. dysgalactiae* is also a significant human pathogen increasingly isolated from patients with invasive diseases, such as severe skin and soft tissue infections, necrotizing fasciitis, toxic shock syndrome, bacteremia, endocarditis, pneumonia, meningitis, and osteoarticular infections^3,4,6,8^. The clinical manifestations and incidences of invasive disease are comparable to those of the closely related species *Streptococcus pyogenes* (group A *Streptococcus*, GAS). Among the virulence factors associated with the pathogenesis of streptococcal infections, the cell surface M protein, encoded by the *emm* gene, is a significant factor related to invasive disease, allowing the bacteria to resist phagocytosis and persist in infected tissues. The *emm* genes are also utilized in molecular epidemiology as the most widely used method for typing streptococcal isolates^3^. The *emm* gene typing, multilocus sequence typing analysis (MLST), and pulsed-field gel electrophoresis (PFGE) have been extensively used to characterize and distinguish streptococci of different origins. Accurate characterization and comparison of strains isolated from different hosts are crucial for determining their taxonomic position and for better understanding the pathogenic and zoonotic potential of these bacteria.

Our study aimed to provide a detailed characterization of *S. dysgalactiae* isolates collected from sheep, focusing on their phylogenetic comparison with strains isolated from other animals and humans, as well as the determination of their phenotypic and genotypic antimicrobial resistance (AMR). This research enhances our knowledge of the zoonotic potential and AMR of *S. dysgalactiae* isolates, supporting the One Health concept.

## Materials and methods

### Bacteria used in the study

In total, 17 *S. dysgalactiae* isolates derived from sheep (n = 17) were included in this study. The isolates were recovered from specimens collected during post-mortem examination and preliminary identified to the species level by 16S rRNA gene sequencing as described in the paper by Didkowska et al.^18^. The animals originated from herds in the Małopolska region in Poland. The sources of isolates were lung (n = 11) or mediastinal and tracheobronchial lymph nodes (n = 6). Previously isolated bacteria were stored frozen at -20 °C in tryptic soy broth (Graso Biotech, Poland) supplemented with 20% glycerol (v/v) (Sigma-Aldrich, Steinheim, Germany). For this research purpose, all isolates were cultured on Columbia agar supplemented with 5% (v/v) defibrinated sheep blood (CA, Graso Biotech, Poland) at 37 °C for 24–48 h under aerobic conditions. The Lancefield serological groups were determined by the latex agglutination MICROGEN®Strep test (Graso Biotech, Poland). The reference strain SDSE ATCC®12394 and *Staphylococcus aureus* ATCC®29213 were also used.

### DNA extraction

Genomic DNA was extracted from the 18-20 h-old bacteria culture on CA. The GenElute™ Bacterial Genomic DNA kit (Sigma-Aldrich, Steinheim, Germany) was used with some modifications to the protocol for Gram-positive bacteria. In the first step, several colonies were picked from a CA and suspended into 200 μL of the Gram-Positive Lysis Solution supplemented with lysozyme (60 mg/mL), followed by incubation for 30 min at 37 °C with shaking. Next isolation steps were performed according to the manufacturer’s instruction with a minor modification of the elution step (40 µL of the Elution buffer heated to 65 °C was used). The DNA concentration was estimated spectrophotometrically (NanoDrop, 1000 Spectrophotometer, Thermo Fisher Scientific, USA), and the DNA samples were stored at -20 °C until further analysis.

### Pulsed field gel electrophoresis (PFGE) typing

The PFGE was performed according to the protocol described by Kaczmarkowska et al.^19^. The separation of restriction fragments was carried out in a 1.1% (w/v) agarose gel under the following conditions: a running time of 21 h, a temperature of 14 °C, a voltage gradient of 6 V/cm, an initial pulse time of 1 s and a final pulse time of 30 s. The reference strain, *S. dysgalactiae* subsp. *equisimilis* ATCC®12394, was included in the study as a marker of DNA molecular size. The molecular size of restriction fragments obtained for SDSE ATCC®12394 was assessed by comparison with fragments obtained by in silico digestion (http://insilico.ehu.es/digest/). The Gel Doc™ EZ Imaging System with Image Lab Software (version 5.2.1) (Bio-Rad, Hercules, CA, USA) was used to visualize and photograph agarose gels. Gel images were analysed using the BioNumerics software version 7.6 (Applied Maths, Sint-Martens-Latem, Belgium). The cluster analysis was performed by Unweighted Pair Group Method with Arithmetic Mean (UPGMA) using the Dice similarity coefficient (optimization and position tolerance set at 1%). Isolates were clustered using an 85% homology cut-off, above which isolates were considered closely related and assigned to the same PFGE cluster^20^.

### Multilocus sequence typing (MLST)

All isolates were submitted to MLST analysis. PCR was performed for seven housekeeping genes (*atoB*, *gki*, *gtr*, *murI*, *mutS*, *recP,* and *xpt*) using primers described by McMillan et al.^21^. The reaction mixture (50 µL total volume) contained 25 µL of Phusion High-Fidelity PCR Master Mix with HF Buffer (2 ×) (Thermo Fisher Scientific, USA), 20 pmol of each primer (Eurofins Genomics Germany GmbH, Germany), and 40 ng of DNA template. PCR was carried out using the following conditions: initial denaturation at 95 °C for 5 min, 28 cycles of denaturation at 95 °C for 45 s, annealing at 55 °C (*atoB*, *gki*, *murI*, *mutS* and *recP*) or 52 °C (*gtr* and *xpt*) for 1 min and extension at 72 °C for 1 min. The final extension step at 72 °C for 2 min follows the last PCR cycle. The obtained amplicons were purified using GenElute™ PCR CleanUp Kit (Sigma-Aldrich, Steinheim, Germany) and sequenced in both the forward and reverse directions (Eurofins Genomics Germany GmbH). The sequence data were analyzed using the Chromas Lite version 2.33 (Technelysium Pty Ltd., Australia). Comparing the loci DNA sequences with the *Streptococcus dysgalactiae* PubMLST reference database^22^, a seven-digit allele code and the strain’s sequence types (STs) were determined.

### The *emm* gene typing

The primers and PCR conditions followed the protocol for typing the *emm* gene encoding the M protein^23^. Amplicons separated by electrophoresis in 1.0% (m/v) agarose gel were purified using GenElute™ Gel Extraction Kit (Sigma-Aldrich, Steinheim, Germany) and sequenced using the Sanger technique. The sequence data were analyzed using the Chromas Lite version 2.33 (Technelysium Pty Ltd., Australia). The *emm* type was assigned using the Streptococci Group A Subtyping Request form Blast 2.0 server available on the CDC website^24^. For one representative isolate of each emm type (isolate 147o and 168o), sequences of almost the entire *emm* gene were obtained and analysed using BLASTN 2.16.0 + ^25,26^. DNA sequence was translated into amino acid sequence using Expasy translating tool (https://web.expasy.org/translate/). The nucleotide and predicted amino acids sequences were compared with the sequences from selected (blast searching) *S. dysgalactiae* strains, including SDSD DB49998-05 (CP033163, QGG97815), SDSE GCS2 (CP117289, ABF82013), SDSE UT10236 (DQ522163) and SDSE 74MP large (EU195123). Multiple sequence alignment was performed using the Muscle program. The *emm* gene sequences obtained in the current study were deposited in the GenBank database (accession numbers OR051764 and OR551293 for 168o and 147o isolates, respectively).

### Phylogenetic analysis based on the* ppaC* and* pfl* genes

The *ppaC* (encoding inorganic pyrophosphatase) and the *pfl* genes (encoding pyruvate formate lyase) from the multilocus sequence analysis (MLSA) scheme developed by Bishop et al.^27^ were used for phylogenetic analysis and comparisons with *S. dysgalactiae* strains isolated from human and animal infections. The obtained amplicons were purified using GenElute™ PCR CleanUp Kit (Sigma-Aldrich, Steinheim, Germany) or ExoSAP-IT reagent (Thermo Fisher Scientific, USA) and were sequenced using the Sanger technique (Eurofins Genomics Germany GmbH). The sequence data were analyzed using the Chromas Lite version 2.33 (Technelysium Pty Ltd., Australia). The phylogenetic comparison was based on the concatenated nucleotide sequences of the *ppaC* and *pfl* genes of representative isolates belonging to different pulsotypes and sequences of various streptococcal strains retrieved from the GenBank database. The sequences were trimmed to a region consisting of 552 (*ppaC*) and 351 (*pfl*) base pairs. Sequence alignments were performed using ClustalX 2.1 software. Evolutionary analyses were conducted in MEGA X^28^. A phylogenetic tree was built using the Maximum Likelihood method and General Time Reversible model^29^, and a bootstrap test was performed with 500 replicates^30^.

### Antimicrobial susceptibility testing

All strains were tested for antimicrobial susceptibility using a strip diffusion method with the Liofilchem® MIC Tests Strips (MIC strips) (Liofilchem, Via Scozia, Italy). The following antimicrobials, belonging to six different functional classes, were tested: penicillin G (PEN), amoxicillin with clavulanic acids (AMC) and cefotaxime (CTX) (beta-lactams), gentamicin (GEN) (aminoglycosides), doxycycline (DXT) (tetracyclines), erythromycin (ERY) (macrolides), ciprofloxacin (CIP) (fluoroquinolones) and sulfamethoxazole with trimethoprim in the ratio 1:19 (SXT). The range of concentrations tested for each antimicrobial agent is shown in Table 1. Müeller-Hinton agar supplemented with 5% (v/v) of sheep blood (MHB, Graso Biotech, Starogard Gdański, Poland) were used. A bacterial inoculum with turbidity adjusted to the 0.5 McFarland standard was prepared in NaCl 0.85% medium (BioMerieux) using the colonies obtained from culture on CA plates, incubated at 37 °C for 18–20 h. The results were read as the point of intersection of the elliptical inhibition zone with the MIC scale value on the test strip. The isolates were considered susceptible or resistant according to interpretive criteria defined by the current Antibiogram Committee of the French Microbiology Society (CA-SFM) guideline Vet2023 or Clinical and Laboratory Standards Institute (CLSI) guideline VET01S ED7:2024 (Table 1)^31,32^. *Staphylococcus aureus* ATCC®29213 was used as a quality control strain. The antimicrobial concentrations required to inhibit the growth of 50% (MIC_50_) and 90% (MIC_90_) of the isolates were determined for each antimicrobial agent tested.

**Table 1:** MIC interpretive criteria for the tested antimicrobial agents.

Antimicrobial agents (strip)	Test range (µg/mL)	MIC breakpoints (µg/mL)			Reference (adopted from)
		S ^1^	I ^1^	R ^1^	
PEN^2^	0.002–32	≤ 0.25	> 0.25–16	> 16	CA-SFM Vet2023 ^4^; *Streptococcus* spp.
AMC	0.016–256 ^3^	≤ 0.25/0.12	0.5/0.25	≥ 1/0.5	CLSI VET01S ED7:2024 ^5^; *Streptococcus* spp.
CTX	0.002–32	≤ 2	4	≥ 8	CLSI VET01S ED7:2024; *S. canis*, group G, beta-hemolytic for cefpodoxime
GEN	0.016–256	≤ 250	500	> 500	CA-SFM Vet2023; *Streptococcus* spp.
DXT	0.016–256	≤ 4	> 4–8	> 8	CA-SFM Vet2023; *Streptococcus* spp. for tetracycline
ERY	0.016–256	≤ 1	> 1–4	> 4	CA-SFM Vet2023; *Streptococcus* spp.
CIP	0.002–32	≤ 0.5	> 0.5–2	> 2	CA-SFM Vet2023 and CLSI VET01S ED7:2024; *Streptococcus* spp. for enrofloxacin
SXT (1:19)	0.002–32 ^3^	≤ 2/38	> 2/38 – 8/152	> 8/152	CA-SFM Vet2023; *Streptococcus* spp.

^1^ S, susceptible; I, intermediate; R, resistant.

^2^ PEN, penicillin G; AMC, amoxicillin with clavulanic acids; CTX, cefotaxime; GEN, gentamicin; DXT, doxycycline; ERY, erythromycin; CIP, ciprofloxacin; SXT, sulfamethoxazole with trimethoprim.

^3^ value of the mix scale refers to the first component of the combination.

^4^ Comité de l’antibiogramme de la Société Française de Microbiologie (CA-SFM). Antibiogram Committee of the French Society of Microbiology Guidelines: Recommandations Vétérinaires 2023 [In French], 2023. Available online: https://www.sfm-microbiologie.org/wp-content/uploads/2023/06/CASFM_VET2023.pdf?

^5^Clinical and Laboratory Standards Institute (CLSI) VET01S-ED7:2024 Performance Standards for Antimicrobial Disk and Dilution Susceptibility Tests for Bacteria Isolated From Animals, 7th Edition, January 2024. Available online: lsivet.org/GetDoc.aspx?doc = CLSI%20VET01S%20ED7:2024&xormat = SPDF&src = BB.

### Antimicrobial resistance genetic determinants

Resistant isolates were screened for the presence of horizontally transferable genes commonly associated with tetracycline resistance (*tet*(M), *tet*(O), *tet*(T), and *tet*(K/L)), MLS_B_ resistance (*erm*(A), *erm*(B), *erm*(C), *erm*A(TR)) and trimethoprim resistance (*dfr*(F)). The used primer sets are listed in Additional file 1. The protocols were described in our previous study^33^. The strains with the *tet*(M) gene were also screened for transposons Tn*916*, Tn*5801,* and Tn*5397*, according to previously described^33^. The strains used as positive controls originated from the collection of the Division of Microbiology, Institute of Veterinary Medicine (Warsaw University of Life Sciences, Poland) (*Streptococcus*, *Enterococcus* and *Staphylococcus* species).

### Statistical analysis

Confidence intervals were calculated using the online Sample Size Calculator tool^34^.

## Results

All isolates used in the study were beta-hemolytic on sheep blood agar. Ten out of 17 isolates (58.8%, CI95%: 32.9%-81.6%) presented Lancefield group A antigens, while seven isolates (41.2%, CI95%: 18.4%-67.1%) belonged to Lancefield group C (Table 2). The isolates were then subjected to accurate molecular characterization.

**Table 2:** Characteristics of the *S. dysgalactiae* isolates included in this study.

No	Strains	Lancefield group	*emm* type	PFGE pulsotype	ST
1	142o	A	nontypeable	P1	NEW
2	143o	C	nontypeable	P3	564
3	145o	A	stL1376.0	P4	248
4	147o	C	stL2764.0	P2	338
5	148o	A	stL1376.0	P4	248
6	150o	A	stL1376.0	P6	248
7	151o	A	stL1376.0	P11	NEW
8	153o	A	stL1376.0	P10	248
9	154o	A	stL1376.0	P7	248
10	156o	C	stL1376.0	P7	248
11	157o	C	stL1376.0	P8	248
12	162o	A	nontypeable	P3	564
13	168o	C	stL1376.0	P5	248
14	169o	C	stL1376.0	P9	248
15	170o	A	stL1376.0	P4	248
16	177o	A	nontypeable	P3	564
17	182o	C	stL1376.0	P4	248

### PFGE

**Fig. 1:**
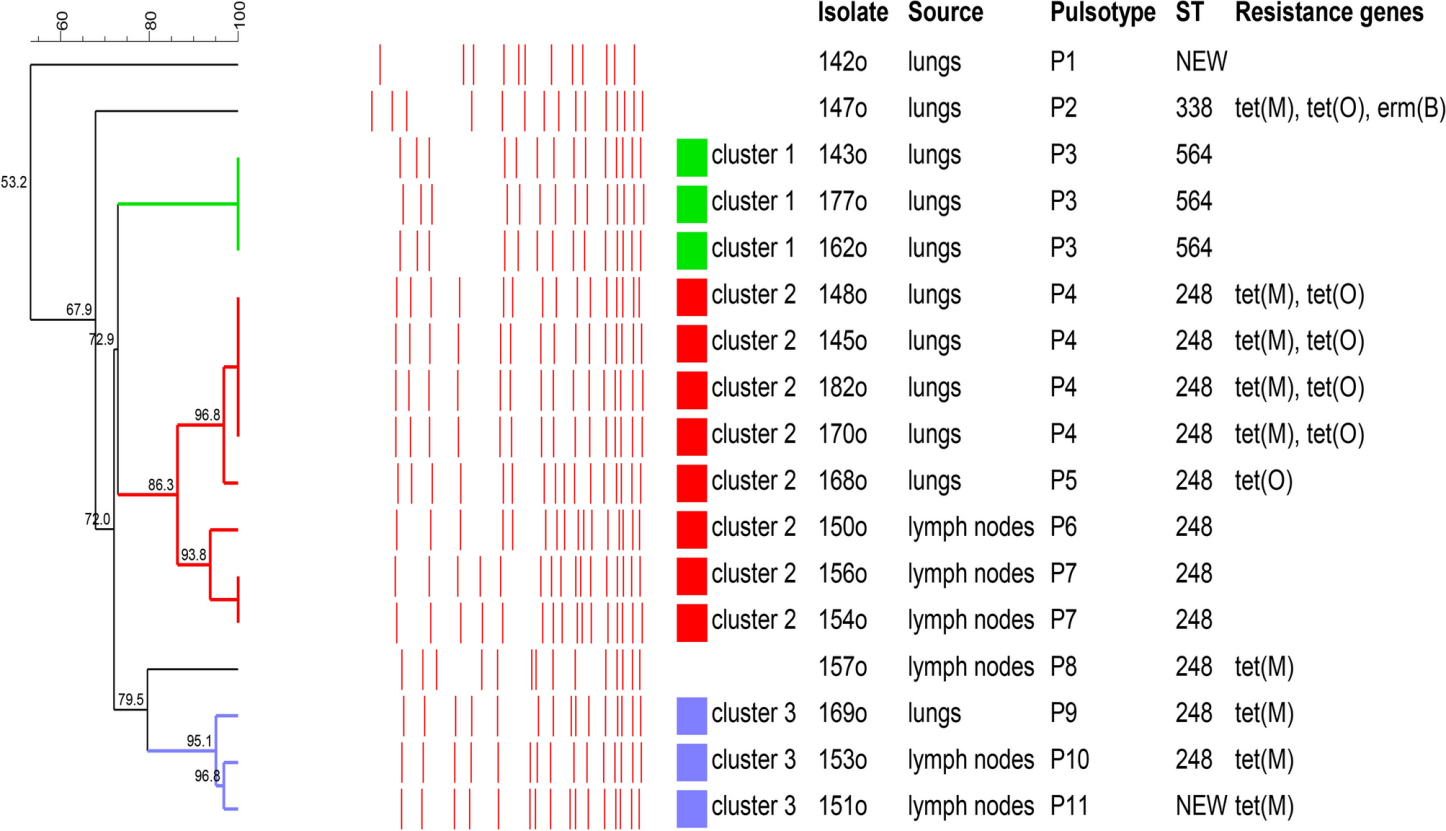
Genetic relatedness of the *S. dysgalactiae* isolates analyzed by PFGE. Dendrogram showing the degree of similarity among 17 tested ovine *S. dysgalactiae* isolates based on the results of PFGE analysis. Red lines indicate the obtained pulsotypes marked P1-P11. Three clusters (colored squares) were defined from groups of closely related isolates sharing at least 85% similarity.

PFGE of chromosomal DNA digested with *Sma*I yielded 12 to 16 fragments in the approximately 20–690 kb size range. Seventeen ovine *S. dysgalactiae* isolates were distributed among 11 distinct PFGE pulsotypes (P1 to P11), and the similarity was between 96.8% and 53.2%. The pulsotype P4 was predominant and was observed in four isolates (23.5%, CI95%: 6.8%-49.9%) (Fig. 1). Pulsotypes P3 and P7 included three (17.6%, CI95%: 3.8%-43.4%) and two isolates (11.8%, CI95%: 1.5%-36.4%), respectively. A single isolate represented pulsotypes P1, P2, P5, P6, P8-P11. The cluster analysis of pulsotypes distinguished three clades, grouping eight (47.1%, CI95%: 23.0%-72.2%) (cluster 2) and three (17.6%, CI95%: 3.8%-43.4%) (cluster 1 and 3) isolates, which shared at least 85 similarities (closely related strains) (Fig. 1). Strains 142o, 147o and 157o had distinct pulsotypes and were not grouped in any distinguished cluster (P1, P2 and P8 pulsotype, respectively). The same pulsotypes were found in the strains of different Lancefield groups (P3, P4, P7). Distinct pulsotypes were observed among isolates belonging to the same Lancefield group, the *emm* type as well as ST (Table 2).

### MLST

Five STs were identified among tested isolates (Table 2). The main ST was ST248, shown in eleven strains (64.7%, CI95%: 38.3%-85.8%). Three strains (17.6%, CI95%: 3.8%-43.4%) belonged to ST564, and one strain to ST338. Moreover, two novel STs were defined. In the case of the 142o strain, analyses showed the presence of a novel allele in the *xpt* locus and the closest match to the 13 allele with two differences, including substitutions at C282 by G and G357 by A. The strain 151o showed novel alleles in the *gki* locus since sequencing chromatograms indicate the closest match *gki*35 (substitutions at C168 by T).

### The *emm* typing

Thirteen of the 17 isolates (76.5%, CI95%: 50.1%-93.2%) were typeable for the antiphagocytic M protein gene, and two different *emm* types were identified, *stL1376* and *stL2764* (Table 2). The type *stL1376* was the most common and was observed in 12 isolates (70.6%, CI95%: 44.0%-89.7%), while *stL2764* was detected in only one isolate (5.9%, CI95%: 0.15%-28.7%) (147o). The type *stL1376* was shared between group A and C isolates. The nucleotide sequences of almost the entire *emm* gene showed the highest similarity to the gene encoding YSIRK-type signal peptide-containing protein in the SDSD DB49998-05 strain (100% identity with 168o isolate) and to the M-protein genes of SDSE strains of human and animal origin (Additional file 2). The alignment of predicted amino acid sequences from analysed strains reveals the variation in size and the number of repeat blocks, as shown in Additional file 3. The first 20 amino acid residues represent the signal peptide. Only sporadic amino acid identity was observed throughout the A-repeat regions, however, a high similarity was observed in the regions encoding the B, C, and D repeats. The M proteins of 147o and 168o isolates have three B-repeats, which show 98.1% and 97.1% identity, respectively, with the corresponding amino acid region of the M protein from the SDSE GCS2 strain. The 168o isolate has two C-repeat segments with 97.1% identity with the SDSE GCS2 strain, whereas in the 147o isolate, the C region is deleted for only one C repeat (Additional file 3). Most isolates showed two fragments of different sizes (approximately 1400 and 1200 bp), however, sequencing of both fragments showed the same *emm* type. In the case of the remaining four isolates, although PCR for the *emm* gene was positive, the chromatograms obtained in sequencing, despite repetitions, were unreadable.

### Phylogenetic analysis based on the *ppaC* and* pfl* genes

**Fig. 2:**
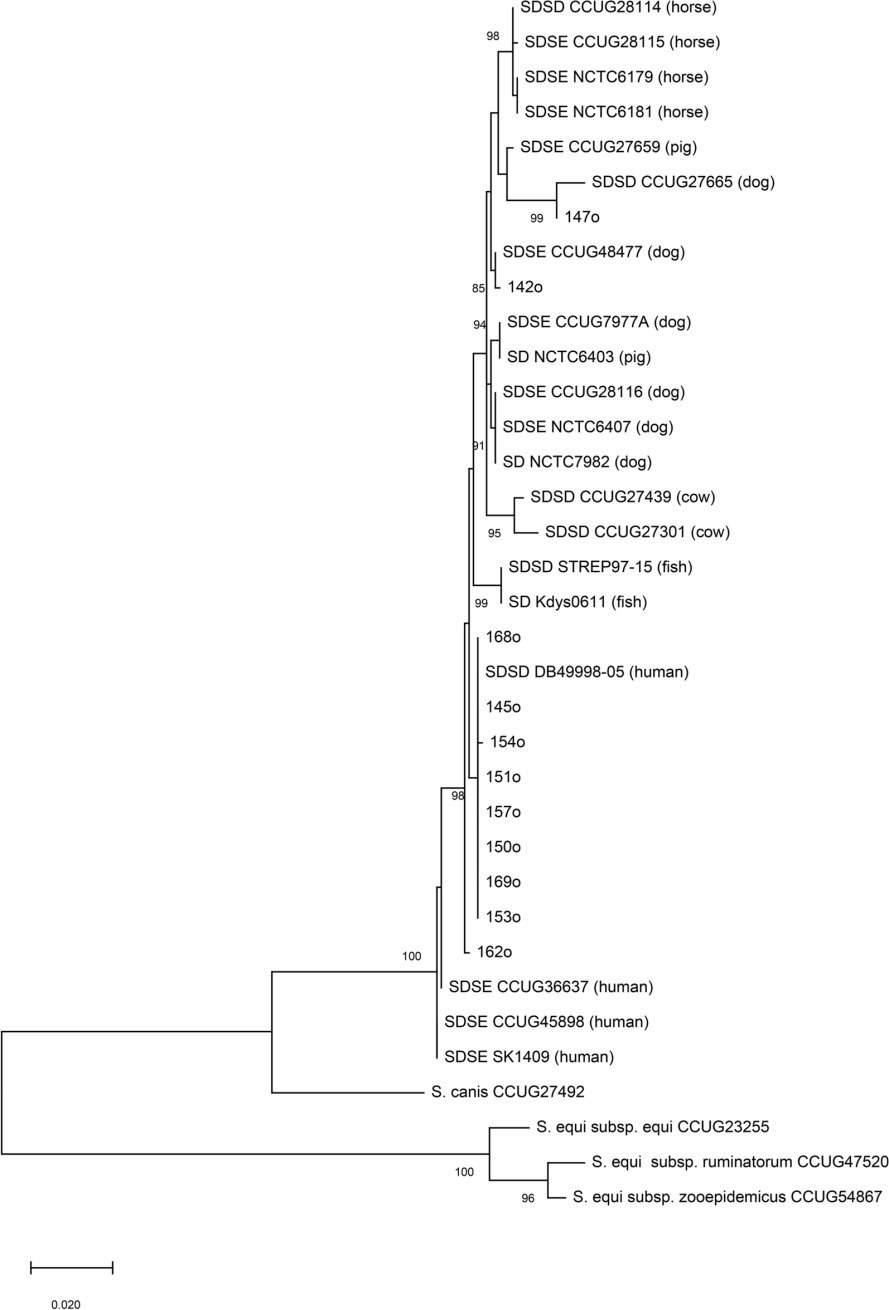
Genetic relatedness of the *S. dysgalactiae* isolates analyzed by the *ppaC* and *pfl* gene sequences. The phylogenetic tree based on concatenated *ppaC* and *pfl* sequences shows the position of selected strains (o indicate tested ovine isolates) and other streptococci, including *S. dysgalactiae* strains isolated from different hosts, *S. equi* and *S. canis*. The evolutionary history was inferred using the Maximum Likelihood method and the General Time Reversible model. The tree with the highest log likelihood (-2786.14) is shown. The concatenated data set consisted of 903 base pairs. Branches clustering sequences with greater than 80% bootstrap support are indicated. Branch lengths correspond to the number of base substitutions per site. The missing isolates were not analysed as they show the same pulsotype.

The phylogenetic tree based on concatenated sequences of the *ppaC* and *pfl* genes is shown in Fig. 2. All analysed *S. dysgalactiae* isolates formed a separated cluster which was divided into two subclusters (with high bootstrap value), the first including only SDSE human isolates and the second one representing all *S. dysgalactiae* isolates derived from different animal hosts and the human strain DB49998-05. Two bovine strains were outgrouped from other strains of animal origin. Notably, most of the sheep-derived isolates from this study were grouped with the human strain DB49998-05, separately from other strains of animal origin. Two isolates (142o and 147o) representing unique pulsotype (P1 and P2) and MLST profile (ST newly described in this paper and ST338) were grouped closely with dog-derived strains.

### Antimicrobial susceptibility testing

The MICs, MIC_50_ and MIC_90_ values of tested antimicrobial agents are shown in Table 3. The MIC ranges for particular antimicrobials were as follows: for PEN < 0.08–0.023 mg/L, for CTX 0.012–0.047 mg/L, for AMC 0.016–0.025 mg/L, for GEN 0.5–1.5 mg/L, for DXT 0.38- > 256 mg/L, for ERY 0.047- > 256 mg/L, for CIP 0.38–4 mg/L and for SXT 0.019- > 32 mg/L (Table 3). According to the used breakpoints (Table 1), four isolates (23.5%, CI95%: 6.8%-49.9%) were susceptible to all tested antimicrobials, including two isolates classified as intermediate to CIP. All 17 isolates were susceptible to all tested beta-lactams (PEN, CTX and AMC) and GEN. The highest frequency of resistance was noted for DXT, since ten isolates (58.8%, CI95%: 32.9%-81.6%) presented MIC values above the breakpoint (MICs > 8). Only one isolate (5.9%, CI95%: 0.15%-28.69%) was resistant to ERY, CIP or SXT. Among 17 isolates, nine displayed resistance to one antimicrobial (52.9%, CI95%: 27.8%-77.0%), and two isolates (11.8%, CI95%: 1.5%-36.4%) were resistant to two antimicrobials, DXT and SXT or DXT and ERY (Table 3). Eight isolates (47.1%, CI95%: 23.0%-72.2%) displayed intermediate resistance to CIP and one isolate to ERY.

**Table 3:** Distribution of MIC, MIC_50_ and MIC_90_ values of antimicrobial agents for tested *S. dysgalactiae* isolates.

Isolate	MIC values (µg/mL)							
	beta-lactams			aminoglycosides	tetracyclines	macrolides	fluoroquinolones	sulfonamides
	PEN^1^	CTX	AMC	GEN	DXT	ERY	CIP	SXT
142o	0.016	0.032	0.023	0.5	0.38	0.047	1*	0.075
143o	0.016	0.032	0.023	0.75	0.75	0.094	1*	0.19
145o	0.016	0.032	0.025	1	64**	0.125	2*	> 32**
147o	0.008	0.032	0.016	0.5	32**	> 256**	0.75*	0.125
148o	0.016	0.032	0.025	1	> 256**	0.19	0.75*	0.25
150o	0.016	0.032	0.023	0.75	0.5	0.064	1*	0.094
151o	0.016	0.023	0.016	0.75	64**	1	0.75*	0.19
153o	0.023	0.012	0.016	0.75	64**	1	0.5	0.38
154o	0.012	0.032	0.023	0.5	0.38	0.064	0.75*	0.125
156o	0.023	0.047	0.023	1	0.5	0.75	0.38	1
157o	0.008	0.032	0.023	0.75	32**	1	0.5	0.19
162o	0.016	0.032	0.023	1	1	0.75	4**	0.38
168o	0.016	0.032	0.016	0.75	16**	1	0.5	0.19
169o	0.023	0.032	0.023	2	48**	1.5*	0.38	0.19
170o	0.008	0.023	0.016	0.5	16**	0.19	0.5	0.125
177o	0.016	0.023	0.023	1.5	0.75	0.38	0.5	0.5
182o	0.012	0.047	0.016	1.5	> 256**	0.19	0.5	0.25
MIC_50_	0.016	0.032	0.023	0.75	16	0.38	0.75	0.19
MIC_90_	0.023	0.032	0.023	1.5	64	1	1	0.5

^1^ Antimicrobial agents used in this study: PEN, penicillin G; AMC, amoxicillin with clavulanic acids; CTX, cefotaxime; GEN, gentamicin; DXT, doxycycline; ERY, erythromycin; CIP, ciprofloxacin; SXT, sulfamethoxazole with trimethoprim.

* MIC value displaying an intermediate phenotype based on the breakpoints defined in Table 1;

** Bolded MIC value displaying a resistant phenotype based on the breakpoints defined in Table 1.

### Antimicrobial resistance genetic determinants

The distribution of the tested acquired AMR genes is presented in Table 4. Eleven isolates (64.7%, CI95%: 38.3%-85.8%) were positive for at least one of the AMR genes. The *tet*(M) and *tet*(O) genes encoding ribosomal protection proteins were found in nine (90.0%, CI95%: 55.5%-99.75%) and six (60.0%, CI95%: 26.2%-87.8%) DXT-resistant isolates, respectively. Five isolates (50.0%, CI95%: 18.7%-81.3%) carried both, the *tet*(O) and *tet*(M) genes. In four *tet*(M)-positive isolates (44.4%) the *xis-Tn* gene of the conjugative transposon Tn*916* was identified. The *tndX* and *int* genes of the Tn*5397* and Tn*5801* conjugative elements, respectively, were not detected. In one macrolide resistant isolate the *erm*(B) gene was identified. All resistant isolates were negative for other tested tetracycline (*tet*(T), *tet*(K), *tet*(L)) and MSL_B_ *erm*A(TR) resistance genes and the *dfrF* gene.

**Table 4:** Distribution of antimicrobial resistance genes identified in the studied *S. dysgalactiae* isolates from sheep.

Isolate	Resistance phenotype	Resistance genotype
142o	[CIP^1^] ^2^	n.d
143o	[CIP]	n.d
145o	DXT, [CIP], SXT	*tet*(M), *tet*(O)
147o	DXT, ERY, [CIP]	*tet*(M), *tet*(O), *erm*(B)
148o	DXT, [CIP]	*tet*(M), *tet*(O)
150o	[CIP]	n.d
151o	DXT, [CIP]	*tet*(M), Tn*916*
153o	DXT	*tet*(M), Tn*916*
154o	[CIP]	n.d
157o	DXT	*tet*(M), Tn*916*
162o	CIP	n.d
168o	DXT	*tet*(O)
169o	DXT, [ERY]	*tet*(M), Tn*916*
170o	DXT	*tet*(M), *tet*(O)
182o	DXT	*tet*(M), *tet*(O)

^1^CIP, ciprofloxacin; DXT, doxycycline; SXT, sulfamethoxazole with trimethoprim; ERY, erythromycin; CLI, clindamycin.

^2^Square bracket indicates an intermediate phenotype;

*n.d.* not determined.

## Discussion

Despite the emerging role of *Streptococcus dysgalactiae* in animal and human infections, the pathogenicity, zoonotic potential and drug resistance of this bacterium are still poorly understood. In particular, literature data regarding strains isolated from animals are scarce. To date, only two papers have undertaken in-depth studies of ovine *S. dysgalactiae* isolated in Norway^12,15^. In this research, we characterized *S. dysgalactiae* isolated from the lung or mediastinal and tracheobronchial lymph nodes of sheep in Poland by Didkowska et al.^18^. The isolates were analyzed using MLST, *emm* typing, and sequencing of the *pfl* and *ppaC* genes from the MLSA scheme. These methods are the most commonly used typing tools for the genetic characterization and molecular epidemiology of GAS (Group A streptococci) and *S. dysgalactiae* infections, enabling comparison with previously described strains from human and animal infections^2,4,7,21,35–38^.

The studied isolates belonged to the Lancefield groups A and C. According to literature data, group C is the most predominant serogroup in swine (100%)^35^ and equine *S. dysgalactiae* isolates (88.9%)^7^, while group L was found in 11.1% of equine isolates^7^. Also, the PubMLST database for *S. dysgalactiae* indicates that group C is the most common among *S. dysgalactiae* isolates of animal origin^39^. In *S. dysgalactiae* strains with specified serogroup, C antigen was present in 100% (23/23), 89% (99/111), 89% (24/27), and 79% (19/24) of strains isolated from cattle, horses, pigs, and dogs, respectively. The antigen L was noted in 17% (4/24), 11% (12/111), and 11% (3/27) of strains isolated from dogs, horses, and pigs, respectively. Group A has been previously recorded only in the case of one canine *S. dysgalactiae* isolate^39^. The criteria described by Vieira et al. do not include *S. dysgalactiae* strains with the Lancefield group A surface antigen since *S. dysgalactiae* subspecies *equisimilis* strains have been classified to Lancefield groups C, G, or L, and *S. dysgalactiae* subspecies *dysgalactiae* strains to serogroup C^1^. It has been assumed that *S. dysgalactiae* received the group A antigen through homological recombination between the ancestral group C SDSE strain and *Streptococcus pyogenes* belonging to group A (GAS)^40^*.* As a result, a chromosomal segment including several structural genes contained in the 12-genes group A carbohydrate biosynthetic cluster (*gac*A-L cluster) was transferred^40^. It should be highlighted that the Lancefield group A carbohydrate is not only a structural component of the cell wall but also an important virulence determinant of streptococci. Van Sorge et al.^41^ showed that the Lancefield group A antigen is a polyrhamnose backbone with N-acetylglucosamine side chains (GlcNAc), which play a crucial role in GAS pathogenesis, increasing bacterial survival and resistance to host innate immunity^41^. Thus, finding the group A antigen in 10 out of 17 tested isolates suggests the presence of the *gac* locus containing genes directly involved in the biosynthesis of GlcNAc affecting streptococcal virulence. SDSE strains belonging to the A serogroup, isolated from invasive human infections, are increasingly described in the literature^40,42^. To the best of our knowledge, among the animal-derived *S. dysgalactiae* strains, Lancefield’s group A has been previously noted only in *S. dysgalactiae* isolates from dog^22^ and pigs^19^, thus this is the first study reporting ovine *S. dysgalactiae* isolates belonging to the serogroup A.

Another factor that plays an important role in the pathogenesis of streptococcal infections is the surface M-protein, a multifunctional molecule with antiphagocytic activity, affecting the ability to host cell adhesion and invasion and evasion of the host immune system^43,44^. The M-protein is a key virulence determinant of *S. pyogenes* and is present in all isolates^45^. Homologs of this protein are also consistently found in human SDSE strains. In contrast, the *emm* gene encoding the M-protein is recognized less frequently in animal-associated strains. Specifically, the *emm* gene was amplified in only one (9%) swine isolate and 60 (56%) equine *S. dysgalactiae* isolates^7,35^. However, the same *emm* type was recognized in both animal and human *S. dysgalactiae* or even *S. pyogenes* strains, indicating the possibility of gene acquisition through horizontal gene transfer^7,46^. The *emm* typing has proved to be of particular importance in analyzing the molecular epidemiology of GAS and SDSE human infections^3,36–38,40,46^.

In the previous study, including ovine *S. dysgalactiae* isolates, *emm*-like genes with very high homology to SDSE and *S. pyogenes* M-protein genes were identified. However, they were assigned to different new *emm*-types^12^. In contrast, the *emm* genes detected in our isolates were typed as *stL2764* and *stL1376*. Both these *emm* types had been previously identified among animal as well as human *S. dysgalactiae* strains^6,7,35,47^. Almost the entire sequence of the *stL1376 emm* gene from the 168o isolate showed 100% identity to the gene encoding YSIRK-type signal peptide-containing protein of the SDSD DB49998-05 strain. In fact, the arrangement of the protein encoded by this gene (QGG97815) closely resembles that found in the SDSE M protein. It includes the YSIRK signal peptide, regions representing the A, B, C, and D repeats, and a transmembrane LPxTG anchor domain (Additional file 3). Interestingly, the SDSD DB49998-05 strain was isolated from a human blood sample and described as the first case of SDSD zoonotic infection^6,48^. Moreover, the *stL1376* type was also recognized in other *S. dysgalactiae* strains isolated from human blood, acute glomerulonephritis, and skin^6,36–38^. In contrast, the *stL2764* was shown previously in *S. dysgalactiae* strains isolated from humans, horses, pigs, dogs, and lizard^2,7,35,46,47^.

The ovine strains characterized by Porcellato et al.^12^ and Smistad et al.^15^ represented five MLST types (ST454, ST531, ST245, ST298 and ST306), some of which (ST245, ST298, ST306) were previously exclusively identified in isolates of bovine origin. Two STs (ST454 and ST531) dominated among ovine isolates. In contrast, the STs found in most of our isolates were previously described in human and swine strains. Most of the isolates belonged to ST248, detected previously in the non-invasive SD22 strain recovered from the skin and soft tissue in humans^37^. Interestingly, the pan-genome analysis showed that the SD22 strain groups within a clade of different strains of animal hosts and separates from the clade grouping most strains recovered from human infection. It suggests a possible case of zoonotic infection^37^. Moreover, ST248 shares six allelic loci with ST341, previously ascribed to the human-derived SD DB49998-05 and DB60705-15 strains, indicating their close relationship. The other detected ST338 and ST564 were found previously in *S. dysgalactiae* strains from the peritoneal cavity and joint of pig and chicken, respectively. This analysis is in line with the results of phylogenetic analysis based on the *ppaC* and *pfl* gene sequences. Our isolates clustered in a clade containing strains derived from different animal hosts (dogs, pigs, fish, and horses), however they clearly separated from the bovine strains and from the human strains clade. Moreover, the analysed *ppaC* and *pfl* sequences of most tested isolates had 100% identity with human *S. dysgalactiae* invasive strain DB49998-05 sequences. Other Authors obtained similar results based on the MLSA scheme^2,7^. Moreover, Jensen and Kilian^2^ showed that phylogenetic trees based on concatenated seven housekeeping genes, as well as only *ppaC/pfl* genes, were consistent.

*S. dysgalactiae* subspecies’ definition remains unclear and is the subject of ongoing debate. Based on genetic comparisons, recent studies have shown that *S. dysgalactiae* species strains segregate into two clearly distinct clades, which correspond to both subspecies. The subspecies *equisimilis* includes only strains of human origin, while subspecies *dysgalactiae* includes strains from various animal hosts, including more phylogenetically distinct bovine strains^2,7,8^. The ovine strains presented in our study are clearly grouped in a clade that includes strains from animals other than cattle. In contrast, Porcellato et al.^12^ showed, based on a pangenome analysis, that bovine and the majority of ovine isolates were almost identical and formed a tight phylogenetic clade, clustered separately from strains of other hosts^12^. Only one ovine strain clustered phylogenetically with porcine isolates^12^. It indicates that sheep isolates are more heterogeneous and may be adapted to different host species, representing a bovine clade as well as a clade of other animal hosts. Nevertheless, further characterization of a larger number of *S. dysgalactiae* isolates from various animal hosts, including whole-genome sequencing, is required for accurate taxonomic delineation of *S. dysgalactiae* species, as well as a better understanding of the host adaptation and clarify the zoonotic potential of this pathogen.

Given that *S. dysgalactiae* strains derived from animals are potential zoonotic agents, it is essential to determine their phenotype and genotype of antimicrobial resistance. Nevertheless, data on antimicrobial susceptibility, including genetic resistance determinants of streptococci, are limited. In this study, beta-lactam antibiotics were highly active among antimicrobial agents against tested isolates, and similar results were obtained by other Authors^4,19,35,49–51^. However, some studies have reported a high rate of beta-lactam resistance in *S. dysgalactiae* isolates associated with the gene *blaZ*, *blaTEM*, *blaIMP,* and *blaSPM-1*^17,52^. In contrast, DXT showed the highest MIC_50_ and MIC_90_ values, and these results were consistent with previous studies that revealed high tetracycline resistance among *S. dysgalactiae* strains worldwide, ranging between approximately 30%-90%^4,19,35,49–53^. Moreover, high tetracycline resistance has also been observed in other streptococci species^33^. In the tested *S. dysgalactiae* isolates, resistance to DXT was due to the presence of the *tet*(M) and *tet*(O) genes, and this is consistent with the data regarding the resistance in streptococci of both human and animal origin^4,19,33,35,50–53^. *S. dysgalactiae* also harbors other genes associated with tetracycline resistance, such as *tet*(K), *tet(*L), *tet*(*S*), and *tet*(T)^4,17,50–53^. In four isolates, the *tet*(M) gene was linked with the Tn*916* transposon, and the acquired resistance facilitated by mobile genetic elements through horizontal gene transfer poses a greater threat due to easy dissemination.

The low MIC values were observed for ERY and SXT since only single isolates were resistant. The macrolide phenotype was associated with the presence of *erm*B. In other studies, the resistance of streptococci to macrolides was observed at various frequencies, from approximately 10%^4,19,50^ to even 70–80%^35,53^. Most resistant streptococci carried *erm*(B), *erm*(C), *erm*A(TR) variant, or *mef*(A/E) genes^4,17,35,49,50,52^. Nikolasein et al. reported a high rate of susceptibility to SXT [49,]. On the other hand, other Authors noted the higher frequency of resistance to SXT/sulfonamide in human SDSE isolates and SDSD isolates from cases of bovine mastitis, approximately between 9.5% and 83%, respectively^51,52,54^. The resistance phenotype was due to *dfrF* or *sul1* and *sul2* genes^51,52^. Moreover, the *dfrG* gene has also been detected in *S. pyogenes*^55^*.*

The MICs of CIP indicated a lower sensitivity of the isolates to fluoroquinolones (FQs). Reduced susceptibility to CIP (MIC 1 µg/mL) was also noted in 10 out of 30 (33%) tested mink isolates^49^. Furthermore, for enrofloxacin, most porcine isolates (10 out of 11, 91%) showed MIC values ranging from 1 to 2 µg/mL^35^. Pinho et al.^56^ reported a high proportion of levofloxacin-resistant (MICs > 2 µg/mL) SDSE human isolates (42 out of 314, 13%), which was associated with spontaneous mutations in *gyrA*, *parC,* or both genes. Generally, the results of antimicrobial susceptibility testing for FQs are challenging to interpret because various agents and breakpoint interpretative criteria are used. Moreover, studies reporting on FQs resistance mechanisms in streptococci are scarce. Fluoroquinolone resistance is multifactorial and complex to clarify, requiring screening for alterations (point substitutions) in the quinolone resistance determining region (QRDR) of target genes. Additionally, the expression of a quinolone efflux pump can confer low-level resistance to FQs in some streptococci^56,57^. However, data concerning the prevalence of mutations in *S. dysgalactiae* species are scarce^35,56^.

## Conclusion

Most of the literature has focused on the characteristics of *S. dysgalactiae* animal strains isolated from cattle, horses, and pigs. Our study and previous data from Porcellato et al.^12^ indicate that ovine isolates constitute a heterogeneous group and may be adapted to different host species, clustering within a bovine clade and a clade associated with other animal hosts. Regarding the ongoing confusion over the taxonomic division of *S. dysgalactiae* into subspecies, the presented genetic analysis based on *ppaC* and *pfl* genes indicates that the subspecies SDSE is related to human infections, while isolates from animals belong to the subspecies SDSD. For the first time, in this study, we report that ovine isolates display an MLST type identical to those previously found only in human isolates (ST248)^22,37^. Moreover, these ovine isolates showed close relatedness to the human invasive SDSD strain DB49998-05, as indicated by a similar ST (sharing six out of 7 alleles) and identical sequences of the *ppaC, pfl,* and *emm* genes. These methodologies have proven particularly important and are widely used in the molecular epidemiology of beta-hemolytic streptococcal infections. The genetic relatedness of the tested isolates to strains causing human infections suggests that sheep *S. dysgalactiae* isolates should be considered potential zoonotic pathogens.

## Supplementary Information

Supplementary Information.

## Data Availability

Data is provided within the manuscript or supplementary information files.
