# Supplementary material for: Genetic analysis reveals the genetic diversity and zoonotic potential of Streptococcus dysgalactiae isolates from sheep
Source: Sci Rep. 2025 Jan 25;15:3165. doi: 10.1038/s41598-025-87781-3 (PMC11763030; doi:10.1038/s41598-025-87781-3)
Supplement: Supplementary file 1 — Supplementary Information. [file 41598_2025_87781_MOESM1_ESM.pdf]

Supplementary Table 1. The characteristics of primers used for antimicrobial resistance gene detection.

Target	Primer sequence (5'-3')	Annealing temp. (°C)	Amplicon size (bp)	Reference[1]
***tet*(M)**	TTAAATAGTGTTCTTGGAG<br>CTAAGATATGGCTCTAACAA	54	656	Nawaz et al., 2011
***tet*(O)**	GGCGTTTTGTTTATGTGCG<br>ATGGACAACCCGACAGAAGC	50	559	Gibreel et al., 2004
***tet*(T)**	GGCGATGTCATTRCAGAAAAAG<br>AGTGCTTCTATCAGTTTGCTTC	56	521	Stefańska et al., 2022
***tet*(K)/*tet*(L)**	CCTGTTCCCTCTGATAAA<br>CAAACTGGGTGAACACAG	50	1050	Pang et al., 1994
***erm*(A)**	TCTAAAAAGCATGTAAAAGAA<br>CTTCGATAGTTTATTAATATTAGT	52	645	Toomey et al., 2010
***erm*(B)**	GAAAAGGTACTCAACCAAATA<br>AGTAACGGTACTTAAATTGTTTAC	52	639	Toomey et al., 2010
***erm*(C)**	TACAAACATAATATAGATAAA<br>GCTAATATTGTTTAAATCGTCAAT	52	642	Toomey et al., 2010
***ermA*(TR)**	CTTGTGGAAATGAGTCAACGG<br>TTGTTCATTGGATAATTTATC	48	551	Pihlajamäki et al., 2002
dfr(F)	TTAACAACGGGTAATGTGGT<br>AAATAGTCCATATCCACCAG	52	201	Cattoir et al., 2009
**Tn*916*-like *(xis-Tn)***	GCCATGACCTATCTTATA<br>CTAGATTGCGTCCAA	39	476	Agersø et al., 2006
**Tn*5397*-like *(tndX)***	ATGATGGGTTGGACAAAGA<br>CTTTGCTCGATAGGCTCTA	46	610	Agersø et al., 2006
**Tn*5801*-like *(int)***	CCGATATTGAGCCTATTGATGTG<br>GTCCATACGTTCCTAAAGTCGTC	58	722	de Vries et al., 2009

[1] Nawaz, M. *et al.* Characterization and transfer of antibiotic resistance in lactic acid bacteria from fermented food products. *Curr Microbiol* **62**,1081-1089 (2011).

Gibreel, A. *et al*. Incidence of antibiotic resistance in *Campylobacter jejuni* isolated in Alberta, Canada, from 1999 to 2002, with special reference to *tet*(O)-mediated tetracycline resistance. *Antimicrob Agents Chemother* **48**, 3442-3450 (2004).

Stefańska, I., Kwiecień, E., Kizerwetter-Świda, M., Chrobak-Chmiel, D. & Rzewuska, M. Tetracycline, macrolide and lincosamide resistance in *Streptococcus canis* strains from companion animals and its genetic determinants. *Antibiotics (Basel)* **11**,1034 (2022).

Pang, Y., Bosch, T. & Roberts, M.C. Single polymerase chain reaction for the detection of tetracycline-resistant determinants Tet K and Tet L. *Mol Cell Probes* **8**, 417-422 (1994).

Toomey, N., Bolton, D. & Fanning, S. Characterisation and transferability of antibiotic resistance genes from lactic acid bacteria isolated from Irish pork and beef abattoirs. *Res Microbiol* **161**, 127-135 (2010).

Pihlajamäki, M. *et al*. Ribosomal mutations in *Streptococcus pneumoniae* clinical isolates. *Antimicrob Agents Chemother* **46**,654-865 (2002).

Cattoir, V., Huynh, T.M., Bourdon, N., Auzou, M. & Leclercq, R. Trimethoprim resistance genes in vancomycin-resistant *Enterococcus faecium* clinical isolates from France. *Int J Antimicrob Agents* **34**, 390-392 (2009).

Agersø, Y., Pedersen, A. G. & Aarestrup, F. M. Identification of Tn5397-like and Tn916-like transposons and diversity of the tetracycline resistance gene *tet*(M) in enterococci from humans, pigs and poultry. *J Antimicrob Chemother* **57**, 832-839 (2006).

de Vries, L. E., Christensen, H., Skov, R. L., Aarestrup, F.M. & Agersø, Y. Diversity of the tetracycline resistance gene *tet*(M) and identification of Tn916- and Tn5801-like (Tn6014) transposons in *Staphylococcus aureus* from humans and animals. *J Antimicrob Chemother* **64**, 490–500 (2009).

Supplementary Table 2. The comparison of nucleotide sequences of the *emm* gene from tested isolates with the selected sequences from the GenBank database.

NCBI Accession number	Description of the gene	Strain (isolation source)	Results of analysis (nBLAST)
			Identity / Query cover
CP033163	gene encoding YSIRK-type signal peptide-containing protein	*S. dysgalactiae* subsp. *dysgalactiae* DB49998-05 (human, blood)	100% / 100% (168o) [1]<br>97.51% / 72% (147o) [2]
EU195123	*emm*-like gene, M protein	*S. dysgalactiae* subsp. *equisimilis* 74MP large (dog, pharynx)	99.20% / 73% (147o) [2]
DQ522163	*emm* gene, M protein	*S. dysgalactiae* subsp. *equisimilis* UT 10236 (human, blood)	95.28% / 64% (147o) [2]<br>89.43% / 62% (168o) [1]
CP117289	*emm* gene, M protein	*S. dysgalactiae* subsp. *equisimilis* GCS2 (human, throat swab)	

[1]NCBI Accession number OR051764), [2]NCBI Accession number OR551293

Supplementary Figure 1. Multiple alignments of the deduced amino acid sequences of M proteins.

```
                                            signal peptide              mature protein

SP M6             -------------------ASVAVALSVLGAGLVVNTNEVSARVFPRGTVENPDKARELL
SDE GCS2          -------------------ASVAVALSVLGAGLASQT-EVKAEENERLRQAKEQALQEVI
SD 147o           ---------------------VAVALCVLGAGLVVNTNEVGALTITRSMAKDPEKLKALA
SD 168o           ---------------------VAVALCVLGAGLANQT-EVKAEENPRVTEARAEVIAEML
SDSD DB49998-05   MARENTNKHYSLRKLKKGTASVAVALCVLGAGLANQT-EVKAEENPRVTEARAEVIAEML
                                       *****.******.  :*  **  *    *       :  :   :


SP M6             NK-----YDVENSM---------------------LQANNDKLTTENNNLTDQN------      A1, A2
SDE GCS2          DQ-----MPYDHLK--NTLAGAFRKNQEN-------ERDLKDLNDALNNTILENIKSSNQ
SD 147o           ET-----YEVENHK---------------------LTNENGKLTSENDKLTSEN------      A1, A2
SD 168o           RNNNFYGYPFGHLQFGNLLSSTIRDNNNLKETLNKKEEEVGTLLGELDKIVKKNIESGDK      A1
SDSD DB49998-05   RNNNFYGYPFGHLQFGNLLSSTIRDNNNLKETLNKKEEEVGTLLGELDKIVKKNIESGDK      A1
                            : :                         :   *  .   ::    :*


SP M6             -----KNLTTE-NKNLTDQNKNLTT------ENKNLTDQNKNLTTENKE-----------      A2, A3, A4, A5
SDE GCS2          HKKEIGELKSA-NGGLKTALDDMAD------TLNQASRKVNELSAQNKA-----------
SD 147o           -----GKLTSE-NNGLKDSLNSIVD------TLQASSRRASELSAQNKA-----------
SD 168o           YKKEIGQIKAEADAAAQKALDALNNKNEQIAALVDETNTLKEINEQDKNDLETLTKVLDE
SDSD DB49998-05   YKKEIGQIKAEADAAAQKALDALNNKNEQIAALVDETNTLKEINEQDKNDLETLTKVLDE
                       *::.:   :  .  :  :*:  :  :       :*    :.   .*:.  *:*
```

```
SP M6             ------------------LKAEENRLTTENKGLTKKLSEAEEEAANKEREN---KEAIGTLKK     A5, B1
SDE GCS2          ------------------LQAEAE--AAAQKAL-DALNNKNEQIAKLANDNDGLKEAIEGYVD
SD 147o           ------------------LQAEAE--AAAQKAL-DALNNKNEQIAKLANDNDGLKEAIEGYVD
SD 168o           AVAKNIESTNKYKKEIGQLKAEAE--AAAQKAL-DALNNKNEQIAKLANDNDGLKEAIEGYVD     A2
SDSD DB49998-05   AVAKNIESTNKYKKEIGQLKAEAE--AAAQKAL-DALNNKNEQIAKLANDNDGLKEAIEGYVD     A2
                                    *:****  ******* *****************************


SP M6             TLDETVKDKIAKEQE----------SKETIGTLKKTLDETVKDKIAK-EQESK--------     B1, B2, B3
SDE GCS2          TIQQASREVSAKQQELAAAQLQLEAKNTEIEDLKHQ-DNMKSEEIAKLESEAKMLENLIGS     B1
SD 147o           TIQQASREVSAKQQELAAAQLQLEAKNAEIEDLKRQ-DNMKAEEIAKLESEAKMLENLIGS     B1
SD 168o           TIQQASREVSAKQQELAAAQLQLEAKNAEIEDLKRQ-DNMKAEEIAKLESEAKMLENLIGS     B1
SDSD DB49998-05   TIQQASREVSAKQQELAAAQLQLEAKNAEIEDLKRQ-DNMKAEEIAKLESEAKMLENLIGS     B1
                  ***************************:*******.* ****:******************


SP M6             --ETIGTLKKTLDETVKDKIAKEQES----------KETIGTLKKILDETVKDKIAREQK      B3, B4, B5
SDE GCS2          GKRELGDLEAKLADANAQKAKLESEAKMLENLIGSGKREIADLQAKLDEANADKAKLESE      B1, B2, B3
SD 147o           GKRELGDLEAKLADANAQKAKLESEAVMLENLIGSGKREIADLQAKLDEANADKAKLQSE      B1, B2, B3
SD 168o           GKRELGDLEAKLADANAQKAKLESEAVMLENLIGSGKREIADLQAKLDEANADKAKLQSE      B1, B2, B3
SDSD DB49998-05   GKRELGDLEAKLADANAQKAKLESEAVMLENLIGSGKREIADLQAKLDEANADKAKLQSE      B1, B2, B3
                  ************************** ******************************:**
```

```
SP M6               S-----------------KQDIGALKQELAKKDEGNKVSEASRKGLRRDLDASREAKKQV   B5, C1
SDE GCS2            ATILERLLESGKRELAEQQAKLDAANADNAKLTEDKQISEASRQGLRRDLNASREAKKQV   B3, C1
SD 147o             ATILERLLESGKRELAEQQAKLDAANADNAKLTEDKQV----------------------   B3, C1
SD 168o             AAILERLLESGKRELAEQQAKLDAANADNAKLTEDKQVLEASRKRTNRDLEAARDAKKAT   B3, C1
SDSD DB49998-05     AAILERLLESGKRELAEQQAKLDAANADNAKLTEDKQVLEASRKRTNRDLEAARDAKKAT   B3, C1
                    *:***********************************:

                        linker
SP M6               EKDLANLTAELDKVKEEKQISDASRQGLRRDLDASREAKKQVEKALEEANSKLAALEKL   C1, C2
SDE GCS2            EKDLANLTAELDKVKEDKQISEASRQGLRRDLDASREAKKQVEKALEEANSKLAALEKL   C1, C2
SD 147o             --------------------LEASRKRTNRDLEAAREAKKEVDAEL-------------   C1
SD 168o             EAELAETTAKVNKLEEEKQILEASRKRTNRDLEAAREAKKAVDAEL-------------   C1, C2
SDSD DB49998-05     EAELAETTAKVNKLEEEKQILEASRKRTNRDLEAAREAKKAVDAEL-------------   C1, C2
                                         ****:  .***:*:*****  *:   *

SP M6               NKELEESKKLTEKEKAELQAKLEAEAKALKEQLAKQAEELAKLRAGKASDSQTPDA--KPGNK-VVPGKGQAP   D1-D4
SDE GCS2            NKELEESKKLTEKEKAELQAKLEAEAKAL--------------------------------------------   D1
OR551293            -------------------AKLKAEAEAL--------------------------------------------   D1
SD 168o             -------------------AKLKAEAEAL--------------------------------------------   D1
SDSD DB49998-05     -------------------AKLKAEAEALKEQLAKQAEEIAKLKASKEKAPEAPQTPEKPETKPSMPWTALTP   D1-D4
                                       ***:***:**
```

```
SP M6            ---------QAGTKP----NQNKAPMKETKRQLPSTGETANPFFTAAALTVMATAGVAAVVKRKEEN
SDE GCS2         -------------------------------------------------------------------
SD 147o          -------------------------------------------------------------------
SD 168o          -------------------------------------------------------------------
SDSD DB49998-05  ATPIAKDEKKAEVKPAAKANMASTDVKKDEKKLPSTGETVNPFFTAVALAVMATAGVAAVAKRKEEN
                                                                             6 aa charged tail
```

Multiple alignments of the deduced amino acid sequences of M proteins of *S. pyogenes* (SP) strain M6 and *S. dysgalactiae* strain 168o (OR051764), 147o (OR551293), GCS2 (ABF82013) and DB49998-05 (QGG97815). The asterisks designate identities between SD strains, while dots designate charge or hydrophobic conservation.

The signal peptide (red boldface) with YSLRKX$_3$GX$_2$S motif conserved (red frame) and the assumed beginning of the mature protein are indicated. The repeated regions are boxed in different frames to show the different repeats: A repeats are boxed in green, B repeats in blue, C repeats in red, and D repeats in brown. C repeats are separated by 7 aa linker region (pink frame). D repeats region is followed by A proline- and glycine-rich cell wall-spanning region, a hydrophobic membrane anchor region, and a 6-amino-acid charged tail.
